# A functional genetic variant in fragile-site gene *FATS* modulates the risk of breast cancer in triparous women

**DOI:** 10.1186/s12885-015-1570-9

**Published:** 2015-07-30

**Authors:** Fangfang Song, Jun Zhang, Li Qiu, Yawen Zhao, Pan Xing, Jiachun Lu, Kexin Chen, Zheng Li

**Affiliations:** 1 Department of Epidemiology and Biostatistics, Tianjin Medical University Cancer Institute and Hospital, Tianjin, 300060 P. R. China; 2 Department of Biochemistry and Molecular Biology, Tianjin Medical University Cancer Institute and Hospital, Tianjin, 300060 P. R. China; 3 Department of Breast Surgery, Tianjin Medical University Cancer Institute and Hospital, Tianjin, 300060 P. R. China; 4 Key Laboratory of Breast Cancer Prevention and Therapy, Ministry of Education, Key Laboratory of Cancer Prevention and Therapy, Tianjin, National Clinical Research Center of Cancer, Tianjin Medical University Cancer Institute and Hospital, Tianjin, 300060 P. R. China; 5 The Institute for Chemical Carcinogenesis, State Key Lab of Respiratory Disease, Guangzhou Medical University, Guangzhou, 510182 China

**Keywords:** Breast cancer, FATS, Single-nucleotide polymorphism, p53, Parity

## Abstract

**Background:**

The fragile-site associated tumor suppressor (*FATS,* formerly known as *C10orf90*), a regulator of p53-p21 pathway has been involved in the onset of breast cancer. Recent data support the idea that the crosstalk between FATS and p53 may be of physiological importance for reproduction during evolution. The aim of the current study was to test the hypothesis that *FATS* genetic polymorphism can influence the risk of breast cancer.

**Methods:**

We conducted population-based studies in two independent cohorts comprising 1 532 cases and 1 573 controls in Tianjin of North China, and 804 cases and 835 controls in Guangzhou of South China, coupled with functional validation methods, to investigate the role of *FATS* genetic variant in breast cancer risk.

**Results:**

We identified a functional variant rs11245007 (905C > T, 262D/N) in fragile-site gene *FATS* that modulates p53 activation. FATS-262 N exhibited stronger E3 activity to polyubiquitinate p53 than did FATS-262D, leading to the stronger transcriptional activity of p53 and more pronounced stabilization of p53 protein and its activation in response to DNA damage. Case–control studies found that CT or TT genotype was significantly associated with a protective effect on breast cancer risk in women with parity ≥ 3, which was not affected by family history.

**Conclusions:**

Our findings suggest the role of FATS-p53 signaling cascade in suppressing pregnancy-related carcinogenesis and potential application of *FATS* genotyping in breast cancer prevention.

**Electronic supplementary material:**

The online version of this article (doi:10.1186/s12885-015-1570-9) contains supplementary material, which is available to authorized users.

## Background

Breast cancer is both the most common malignancy and the one causing the highest number of cancer deaths in women worldwide. For most sporadic breast cancers, it has been suggested that genetic polymorphisms, especially single nucleotide polymorphisms (SNPs) in low-penetrance susceptibility genes in concert with environmental exposures may be more important. The p53 tumor suppressor protein, through its downstream target p21, plays a key role in sustaining cell-cycle checkpoints after DNA damage to maintain the genomic stability [1, 2]. The defects in this pathway may result in genomic instability and carcinogenesis. Over the past few years, emerging evidence have revealed a role of p53 in regulating human maternal reproduction [3]. It is well-known that reproductive history represents lifetime exposure to hormones and is a significant risk factor for breast cancer, besides the family history of breast cancer [4–9]. However, whether the modulation of p53 activation may contribute to the genetic basis underlying the effect of reproductive history on the risk of breast cancer remains unknown.

Recently, the fragile-site associated tumor suppressor (*FATS,* aka *C10orf90*), a regulator of p53-p21 pathway, has been identified at a common fragile site (CFS) FRA10F mapped to 10q26, a genomic region susceptible to DNA damage and frequently deleted in tumor genomes [10–13]. Our previous reports show that the deficiency of *FATS* mRNA is observed in multiple cancer cell lines and clinically relevant to human cancers including breast cancer [11, 14, 15]. More recently, we reveal that the NH2-terminus of FATS exhibits a unique ubiquitin ligase (E3) activity to promote p53 activation in response to DNA damage [12]. The p53-p21 pathway plays a key role in sustaining cell-cycle checkpoints after DNA damage [1, 2], and the expression of the largest exon of *FATS* is sufficient to activate p53-p21 pathway and suppress tumorigenesis [10–12]. However, whether the non-proteolytic ubiquitination of p53 by FATS may have some physiologic significance and whether the genetic variants of *FATS* may modulate the risk of breast cancer remain unknown.

In this study, we set out to test the hypothesis that *FATS* genetic variant may predispose to breast cancer development using a population-based study design coupled with functional validation.

## Methods

### Study subjects

The study population consisted of two independent cohorts: the Tianjin cohort (Discovery cohort) included 1532 patients with newly diagnosed and histologically confirmed breast cancer and 1573 age-matched (±5 years) healthy female controls, randomly extracted from a population-based case–control study we have set up [16]. As a Replication cohort (Guangzhou cohort), 804 newly diagnosed and histopathologically confirmed breast cancer patients were consecutively recruited between March 1st, 2007 and March 1st, 2011 from four urban hospitals (i.e., the First, the Second and the Tumor Hospitals affiliated to Guangzhou Medical University, and Guangzhou Chest Hospital) and one suburban hospital, Panyu People’s Hospital, with a response rate of 91 %. 835 age-matched (±5 years) healthy controls were recruited from Guangzhou, a city in South China, with a response rate of about 85 %.

All participants enrolled in this study were of Chinese Han ethnicity. The Ethics Committee of Tianjin Medical University Cancer Hospital (TMUCIH) and Guangzhou Medical University (GMU) approved the study protocol, and we obtained written informed consent from all patients and controls to participate in this study.

Each subject filled out a questionnaire about demographics, menstrual and reproductive history, environmental exposures, lifestyle, and family history of cancer. Each subject donated 20 mL of blood that was collected into heparinized tubes and used for DNA extraction and genotyping. Paired normal tissue samples (the distance from tumor tissue: >5 cm) randomly selected from the breast cancer cases included in the study were verified by pathology specialists, and prepared for genotyping and FATS expression quantification. The tissue samples for these selected cases were obtained from the Tissue Bank Facility of TMUCIH with approval of the Institutional Review Board (IRB).

### Quantitative RT-PCR

The total RNA was extracted for cDNA synthesis, and quantitative real-time PCR was performed as described previously [15]. Briefly, Total RNA (5 μg) was transcribed into complementary DNA (Invitrogen kit) and subjected to quantitative RT-PCR analysis. The primers and Taqman probes of FATS (C10orf90) (GenBank accession number: NM_001004298) were 5’-CATTCACATTCCTGGCTGGAGTTA-3’, 5’-CCTCTTGCTGCTTCCAGAAAATACT-3’, and 5’ (FAM)-CAGGGCAGTACACACAAA-(TAMRA)-3’. The primers and Taqman probes of GAPDH were 5’-GAAGGTGAAGGTCGGAGTC-3’, 5’-GAAGATGGTGATGGGATTTC-3’, and 5’(FAM)-CAAGCTTCCCGTTCTCAGCC-(TAMRA)-3’. Assays were carried out using the ABI 7500 TaqMan system (Applied Biosystems). Quantification of FATS gene expression in each sample was determined by measuring PCR cycle number at which the amount of FATS transcripts reached a fixed threshold (C_T_). The average C_T_ value for FATS gene in each sample was obtained from three independent experiments, and normalized by that of GAPDH gene to obtain ΔC_T_. ΔC_T_ = C_T_ (FATS)-C_T_ (GAPDH). The quantity of FATS mRNA in each sample was calculated as 2^−ΔCT^.

### Immunohistochemical analysis

Formalin-fixed and paraffin embedded blocks of 30 breast cancer specimens and paired normal tissues available were analyzed using a rabbit polyclonal FATS (C10orf90) antibody (Abcam, ab122497). Briefly, deparaffinized sections were boiled for 15 min in a 1-mM sodium citrate buffer (pH 6.0) for antigen retrieval. After quenching of endogenous peroxidases with 0.3 % hydrogen peroxide in methanol, followed by two rinses with Tris–HCl buffer, the sections were incubated with the anti-C10orf90 antibody diluted 1:500 overnight at 4 °C. Biotinylated goat anti-rabbit antibody was used as the secondary antibody and developed with liquid DAB substrate chromogen system (Dako). Hematoxylin was used for nuclear counterstaining; the sections were then mounted and coverslipped. The immunohistochemical expression of FATS was evaluated by microscope imaging. Histology analyses were evaluated in a blinded fashion by two pathologists.

### Single nucleotide polymorphism (SNP) identification, selection and genotyping

With reference to the resequencing data of 45 Chinese Han individuals in the International HapMap Project SNP database (http://www.hapmap.org) and National Center for Biotechnology Information (NCBI) dbSNP database (http://www.ncbi.nlm.nih.gov/SNP), we selected twelve SNPs with the minor allele frequency (MAF) > 0.01 reported within the 1.0 kb promoter region, 5’-UTR, coding region, and 3’-UTR of *FATS* (*C10orf90*) gene (Additional file 1). Genomic DNA was extracted from the whole blood using a DNA Blood Mini Kit (QIAGEN), according to the manufacturer’s instructions. These SNPs were validated by DNA sequencing of PCR products in 30 randomly selected healthy subjects (Tianjin cohort). At present study, we preferentially selected one SNP (rs11245007) located in the 3rd coding exon of FATS which is responsible for the major function of FATS protein, with the highest MAF of 0.44 and the most possibly putative functional potential to genotype. Genotyping was performed by using the MGB TaqMan probe assay (Applied Biosystems Inc. [ABI], Foster City, CA). The concordance rate for genotypes was 100 % in 10 % of samples with duplicates.

### Cell culture

The human breast cancer cell line MCF-7 was obtained from the American Type Culture Collection (ATCC) in 2008. The cell line has been last tested and authenticated in 2013 by genetic profiling using the well-known short tandem repeat (STR) loci [17]. The cell line was maintained in culture as an adherent monolayer in DMEM (Invitrogen) medium supplemented with 10 % FBS. Cells were incubated at 37 °C in a humidified atmosphere with 5 % CO2.

### Vectors and site-specific mutagenesis

The expression vector CMV-C10orf90, i.e. FATS-262D, was purchased from Origene. Flag-FATS-262D plasmid was constructed by in-frame inserting full-length FATS cDNA into p3xFlag-myc-CMV-26 vector (Sigma). Flag-FATS-262 N was generated by site-directed mutagenesis, according to manufacturer’s instructions (Stratagene). The primer sequences are 5’-GTCTCAGCAGTGTCCCGATGCCATTTACTATTTGG-3’ and 5’-CCAAAT AGTAAATGGCATCGGGACACTGCTGAGAC-3’. FATS cDNA was in-frame inserted into pGEX-6p-1 vector (GE Healthcare Life Sciences) to generate GST-FATS-262D or GST-FATS-262 N, respectively.

GST-FATS-262 N/ GST-FATS-262D was transformed into Escherichia coli BL21. The bacteria were grown at 30 °C in LB medium, and GST-fusion protein synthesis was induced with 0.5 to 1.0 mmol/L of isopropyl-l-thio-β-D-galactopyranoside. Cells were harvested after 3 to 4 hours. The cell pellet was resuspended in cold sodium-Tris-EDTA lysis buffer [10 mmol/L Tris (pH 8.0), 1 mmol/L EDTA, and 150 mmol/L NaCl] supplemented with lysozyme (1 mg/mL; Sigma) and incubated on ice for 15 minutes. Just before sonication, 1 mmol/L DTT, 10 mmol/L MgCl2, 1 mmol/L phenylmethylsulfonylfluoride (PMSF), and 1 % Sarkosyl (Sigma) were added to the cell lysate and mixed thoroughly. The cell lysate then was sonicated at maximum intensity for 20 seconds. Triton X-100 (2 %) was added, and the cell lysate was mixed gently for 30 minutes to help the fusion protein dissolve. After centrifugation, the GST-fusion protein was purified by Glutathione Sepharose 4B (Amersham Biosciences) and eluted with buffer [50 mmol/L Tris (pH 8.0)] containing 10 mmol/L glutathione (reduced form; Sigma).

MCF-7 cells were transfected with plasmid DNA using a Nucleofector kit (Amaxa) or Lipofectamine 2000 (Invitrogen), according to manufacturer’s instructions.

### Cell fractionation

Cells were washed with cold PBS twice and collected in lysis buffer [20 mmol/L HEPES (pH 7.5), 10 mmol/L KCl, 2 mmol/L MgCl2, 0.5 % NP40, 100 mmol/L NaF, 1 mmol/L Na3VO4, 1 mmol/L PMSF, and 1 % aprotinin]. Let cells sit in lysis buffer on ice for 30 minutes to ensure complete lysis. Spin cells at 12000 g for 10 minutes at 2-4 °C. The supernatant was saved as whole cell lysates.

### Immunoblotting assay

The cell lysates were separated by SDS-PAGE and transferred to PROTRAN nitrocellulose membranes (Schleicher & Schuell, Dassel, Germany). For immunoblot experiments, 20 to 50 μg of lysates in SDS loading buffer were separated by SDS-PAGE. Western blot analysis was exposured to X-ray film for autoradiography.

### Dual luciferase reporter assay

Cells (5 × 10^4^) were transfected with 250 ng of firefly luciferase reporter (pGL3-FATS-luc), 20 ng of the transfection control Renilla vector (pRL-TK) and 100 ng of p53-expressing vector in combination with 500 ng of FATS-expressing vector. At 24 h after transfection, cells were lysed in 1x passive lysis buffer (Promega), and reporter activity was measured using the Dual-Luciferase Reporter Assay System (Promega). Each assay was tested in triplicate in three independent experiments.

### Ubiquitination assay

The ubiquitination assay was performed as described previously [12]. In brief, purified GST-FATS-262 N/GST-FATS-262D (1 μg), E1 (40 ng, Calbiochem or Sigma), and ubiquitin (2 μg, Boston Biochem) was incubated with *in vitro* translated p53 protein in 30 μl reaction buffer (50 mM Tris–HCl [pH 7.4], 2 mM ATP, 5 mM MgCl_2_, 2 mM DTT, 30 mM creatine phosphate, and 0.05 mg/ml creatine phosphokinase) at 30 °C for 90 min. The reactions were stopped by adding 2 x SDS loading buffer and heating at 95 °C for 5 min. Ubiquitinated p53 proteins were detected by immunoblotting using a p53-specifc antibody. In the absence of p53, the assembly of poly-ubiquitin was examined by Western blot using an ubiquitin antibody.

### Statistical analysis

The Kruskal-Wallis H Test was used to analyze the differences of mRNA expression between breast tumor samples and normal breast tissues. A χ^2^ test was used to examine the differences in demographic variables and genotype distribution of *FATS* polymorphisms between patients and controls. A Hardy–Weinberg equilibrium test was performed for the genotype distribution in the controls to evaluate possible selection bias and genotyping errors. The multivariate logistic regression method was used to assess the association between breast cancer risk and *FATS* gene SNP. Odds ratios (ORs) and 95 % confidence intervals (CIs) were calculated with adjustment for known risk factors of breast cancer, such as age, BMI, age at menarche, birth number, duration of breast-feeding, menopause status, oral contraception, smoking status (ever/never), exercise, benign breast disease, and family history of cancer (first/second degree). For cases only, we also performed stratified case-series analysis of the genotype data by clinical phenotypes. All statistical tests were two-sided and a *P* value < 0.05 was considered statistically significant using SAS v9.0 software (Cary, NC, USA).

Heterogeneity of the association between *FATS* SNP and breast cancer risk from the Discovery and Replication cohorts was estimated by the *I*^*2*^ , which was ranked as “no-” (0 % ≤ *I*^*2*^ < 25 %), “moderate-” (25 % ≤ *I*^*2*^ < 50 %), “large-” (50 % ≤ *I*^*2*^ < 75 %) and “extreme-” (75 % ≤ *I*^*2*^ ≤ 100 %) heterogeneity between these two cohorts (Marcos et al., 2009). A random-effects model (DerSimonian and Laird method) or fixed-effects model (Mantel-Hansel method) was used to calculate the pooled OR in the presence (*P* ≤ 0.10) or absence (*P* > 0.10) of heterogeneity, respectively [18, 19]. Analyses were conducted using Stata 11.0.

## Results

### Functional characterization of a genetic variant

**Fig. 1:**
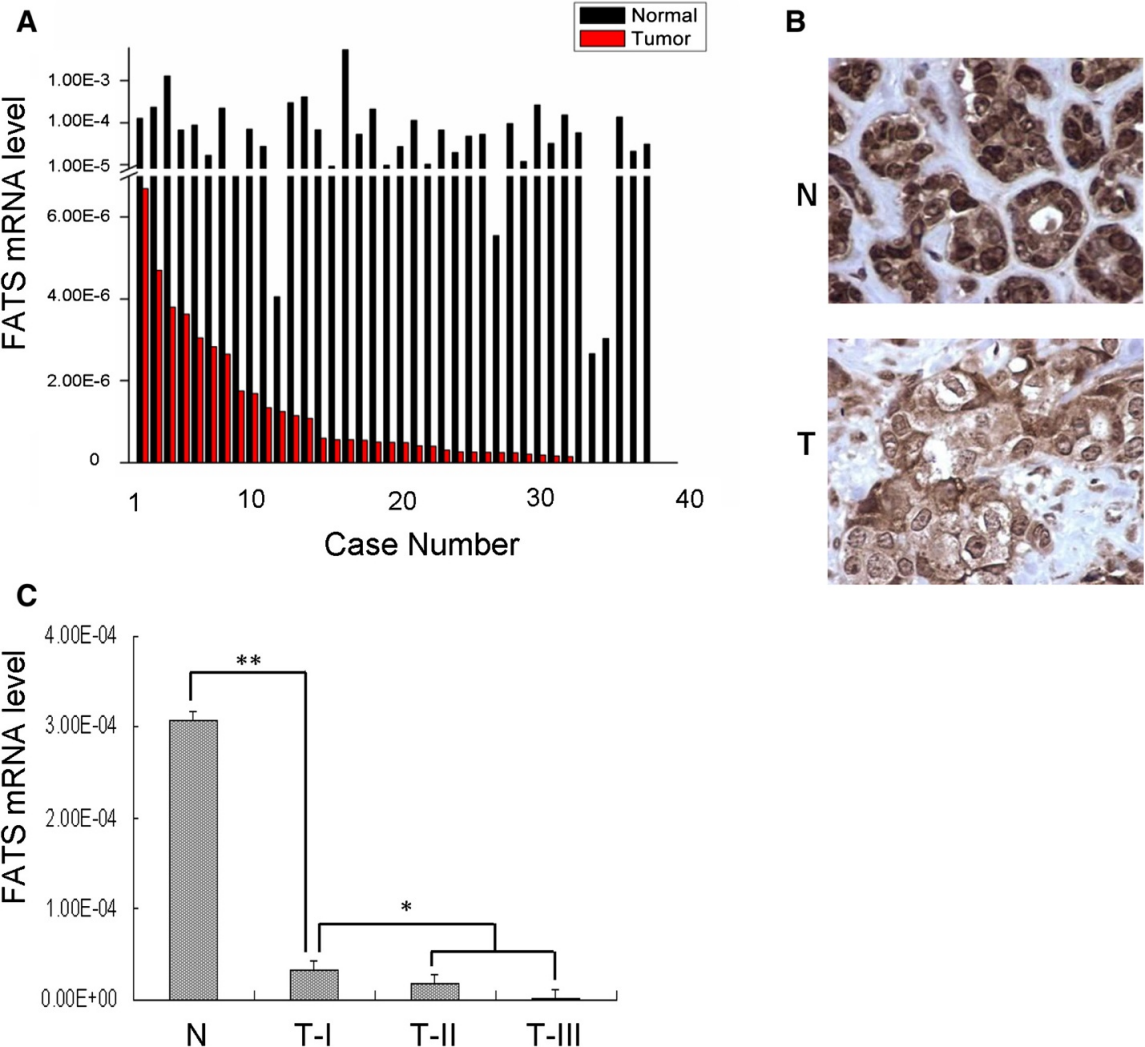
Clinical relevance of *FATS* expression in breast cancer. **a** Quantitative analysis of FATS mRNA (GenBank accession number: NM_001004298) in breast cancer. Tumor samples and paired (>5 cm away) normal breast tissue samples (n = 38) from the Tianjin cohort were subjected to RNA extraction and subsequent RT-PCR analysis. The average C_T_ value for FATS gene in each sample was obtained from three independent experiments, and normalized by that of GAPDH gene to obtain ΔC_T_. The quantity of FATS mRNA in each sample was calculated as 2^−ΔCT^. **b** Immunohistochemistry of FATS in paired human breast tumor samples and normal breast tissue samples (n = 30). A representative picture is shown with the same magnification (40X). N, normal; T, Tumor. **c** The average level of *FATS* mRNA from three independent experiments in breast tumor samples (n = 156) from the Tissue Bank Facility of TMUCIH was profiled according to pathological stages, in comparison with that in normal breast tissues. N: normal (n = 38); T-I: TNM stage I (n = 40); T-II: TNM stage II (n = 97); T-III: TNM stage III (n = 19). *, *P* < 0.05; **, *P* < 0.01

We first validated the clinical relevance of *FATS* expression to breast cancer. Consistent to the sample set from our previous report [14], we confirmed that the expression level of *FATS* mRNA was silent or down-regulated in 100 % matched breast tumor tissues (n = 38), compared with that in normal tissues (Fig. 1a). Consistently, the expression of FATS protein was downregulated in 73.3 % tested breast cancer samples in comparison to that in normal breast tissues (Fig. 1b). We further measured the mRNA levels of *FATS* in breast tumor tissues (n = 156) and found that the average level of *FATS* mRNA even in pathologic stage–I breast tumors was 10-fold lower than that in normal control (*P* < 0.01, Fig. 1c) and were inversely correlated with pathological stages (extensively downregulated or nearly silent in stage III), suggesting that *FATS* deficiency occurred at the early stage of tumorigenesis. These results, in combination with our previous functional studies on *FATS* [10–12], raised the possibility that the SNP in *FATS* may modulate the risk of breast cancer.

**Fig. 2:**
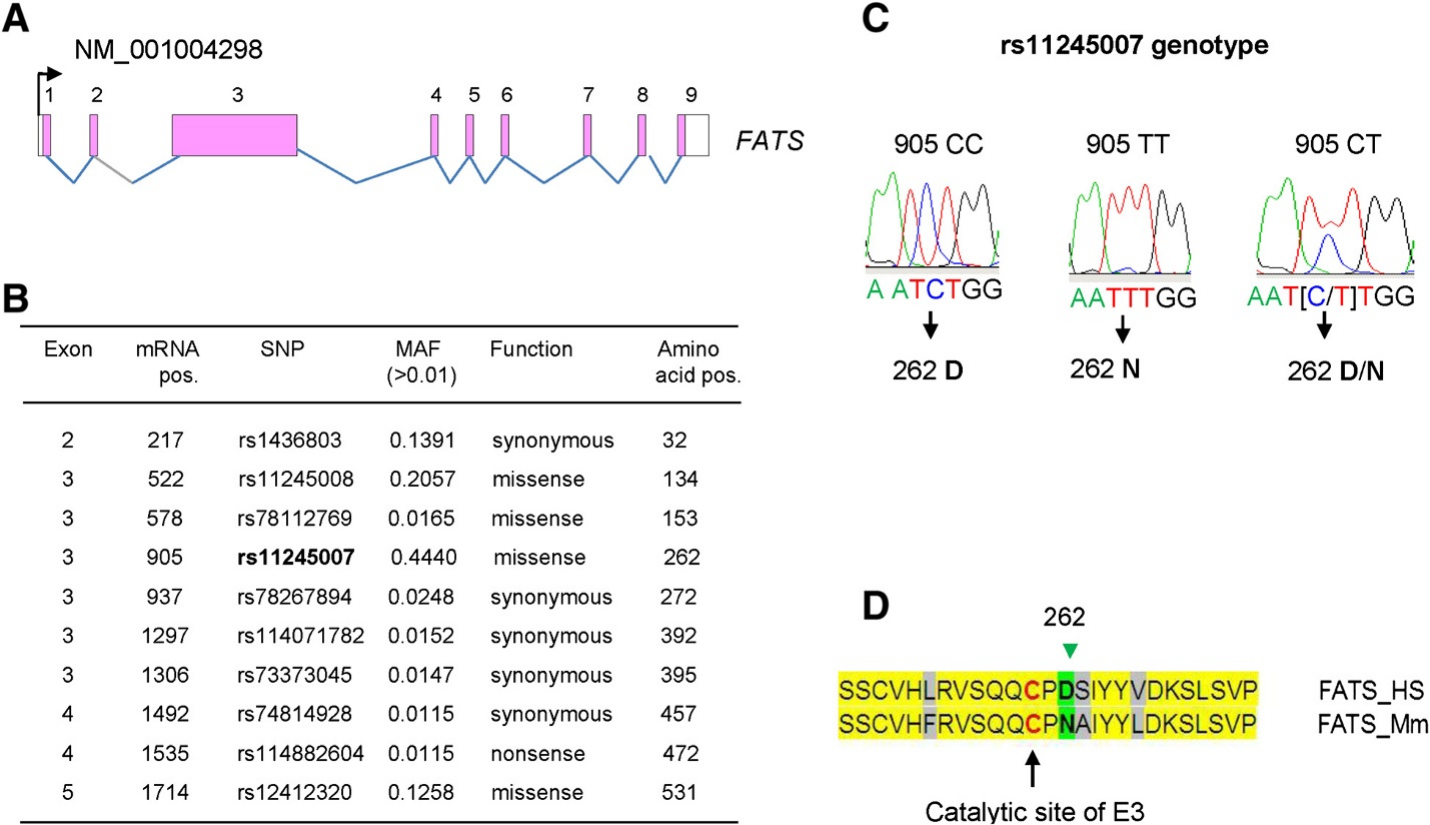
Distribution of major SNP sites in FATS locus and validation of rs11245007. **a** The illustration of genomic organization of human *FATS* locus. **b** The distribution of SNP sites in *FATS* exons. MAF: minor allele frequency. **c** Validation of rs11245007 genotypes in Chinese population. **d** Sequence alignment of amino acid residues within RING/HECT hybrid domain between human FATS and mouse FATS. The conserved catalytic Cys residue of FATS as a unique E2-independent E3 is highlighted

**Fig. 3:**
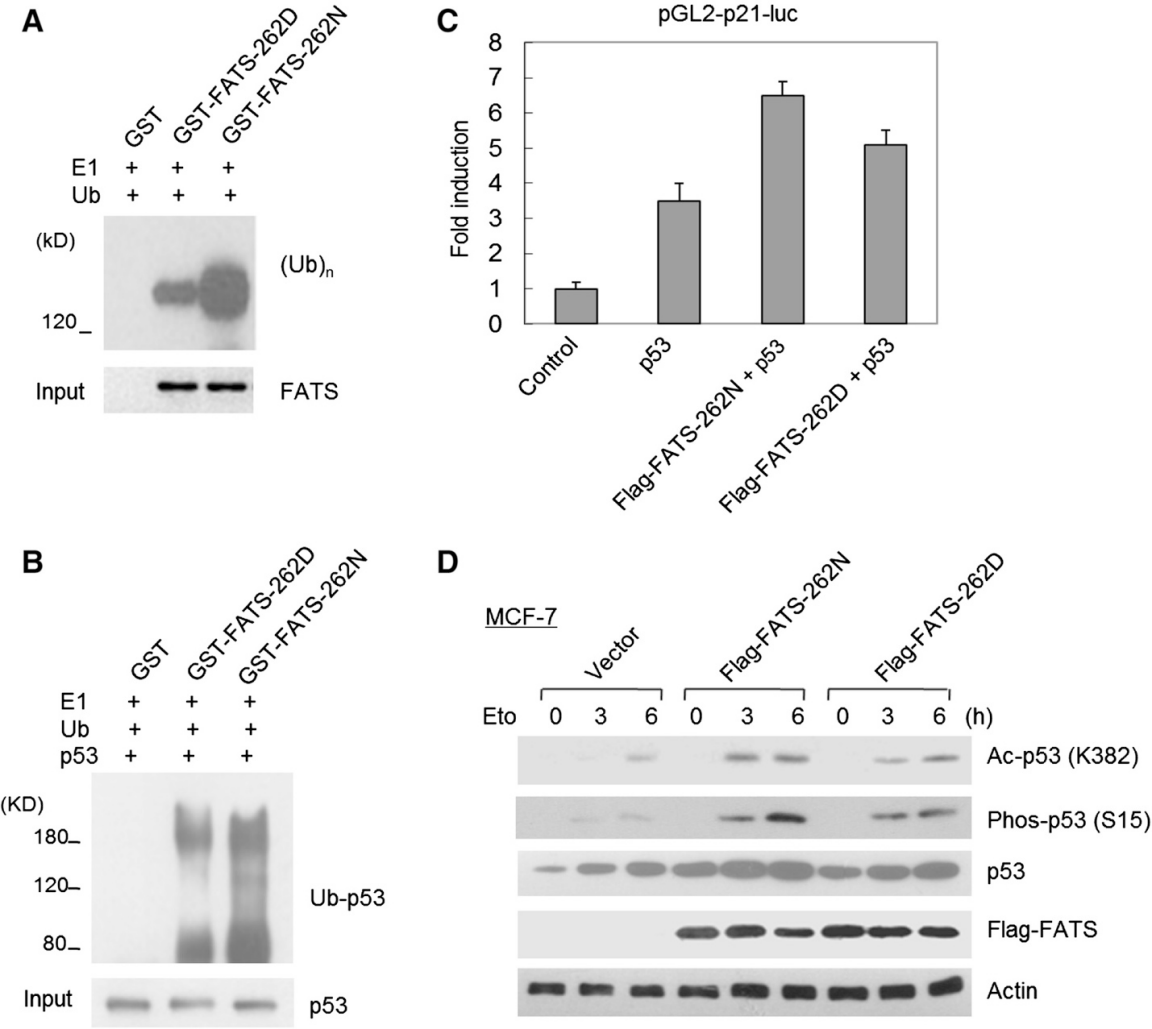
The effects of FATS-262D/N on p53 ubiquitination and activation. **a** Purified GST protein or GST-tagged FATS protein was incubated with purified E1 and ubiquitin protein in ubiquitination buffer at 30 C for 90 min. The polyubiquitination was examined by Western blot using an ubiquitin-specific antibody (n = 3). FATS-262 N exhibited stronger E3 activity than did FATS-262D in assembling ubiquitin chains. **b** Purified GST protein or GST-tagged FATS protein was incubated with purified E1, ubiquitin and p53 protein in ubiquitination buffer at 30 °C for 90 min. The non-proteolytic polyubiquitination was examined by Western blot using a p53-specific antibody (n = 3). **c** MCF-7 cells were transfected with indicated vectors. Luciferase reporter assay was performed in triplicate in three independent experiments after transfection for 24 h. The pGL2-p21-luc vector contains a p21 promoter with p53-responsive elements. **d** MCF-7 cells were transfected with Flag-tagged FATS-262D or FATS-262 N or an empty vector, respectively. After 24 h, cells were treated with etoposide (25 μM) for the indicated time. Cell lysates were subjected to immunoblotting, and the results assessed quantitatively (n = 3). Ac, acetylation; Phos, phosphorylation

To test the hypothesis that SNP in *FATS* gene may contribute to the susceptibility of breast cancer, we firstly evaluated those potentially functional SNPs in *FATS* (Additional file 1). Human *FATS* (*C10orf90*) gene contains 9 exons whose coding protein consists of 699 amino acid residues (Fig. 2a). The power to detect genetic effects is dependent on MAF. There are only 4 SNPs in *FATS* exons with MAF > 0.01 and missense function (Fig. 2b). Notably, a SNP with MAF > 0.25 is located at the largest coding exon of *FATS*, which is responsible for the major function of FATS protein [10–12]. This SNP, rs11245007 (905C > T), with a MAF value of 0.4440 and causing 262D/N substitution, was confirmed by DNA sequencing in Chinese Han population (Fig. 2c). Interestingly, 262D/N is only one amino-acid away from the conserved catalytic Cys residue of FATS protein as an E2-independent E3 (Fig. 2D), raising the probability that FATS-262 N may differ from FATS-262D in its E3 activity. Indeed, the results of ubiquitination assay indicated that the E3 activity of FATS-262 N to assemble polyubiquitin chains was significantly stronger than that of FATS-262D in an E2-independent manner (Fig. 3a).

Because FATS-catalyzed non-proteolytic polyubiquitination of p53 is required for robust activation of p53 in response to DNA damage [12], we further investigated whether FATS-262D or FATS-262 N may cause different modification status of p53 polyubiquitination. As shown in Fig. 3b, the polyubiquitination of p53 by FATS-262 N was more pronounced than that of FATS-262D. Consistently, FATS-262 N exhibited a stronger effect on stimulating the transcriptional activity of p53 (Fig. 3c) and facilitating the stabilization of p53 protein in response to DNA damage (Fig. 3d). Meanwhile, the acetylation and phosphorylation of p53, which are detectable after DNA damage and coupled with p53 activation, was more remarkable in the presence of FATS-262 N than in the presence of FATS-262D (Fig. 3d). Therefore, rs11245007 (905C > T, 262D/N) was a functional SNP with impact on p53 activation.

### Population-based analysis of the SNP rs11245007

The functional identification of rs11245007 (905C > T, 262D/N) in *FATS* prompted us to test its genetic effect on breast cancer risk. We performed a case–control study (Discovery Cohort) that included 1532 breast cancer cases and 1573 healthy controls. The study subjects in this cohort were all recruited from Tianjin, a metropolis in North China (Additional file 2). As expected, patients in the discovery set reported a greater number of known risk factors for breast cancer than did the controls. For example, significantly larger proportions of patients than the controls had fewer birth numbers, less breast-feeding time and physical activity, were nulliparous and younger at menarche, and had history of benign breast disease, and family history of cancer. Genotype frequencies among the controls did not show significant departures from Hardy–Weinberg equilibrium (*P* = 0.407). We did not observe a difference in the genotypic frequencies of rs11245007 between patients and controls (*P* = 0.907), and the results of a case-only analysis indicated that the rs11245007 genotypes were not significantly associated with mean age at diagnosis, lymph node metastasis, and expression status of estrogen receptor (ER) or progesterone receptor (PR) (Additional file 3). In Discovery cohort, the rs11245007 TT or TT + CT genotypes did not show a significant protective effect on overall risk of breast cancer, although the CT genotype was associated with a decreased overall risk of breast cancer compared with the CC genotype (OR, 0.796; 95 % CI, 0.638-0.992; *P* = 0.042) (Table 1).

**Table 1:** Overall and stratified analyses on the association of rs11245007 genotypes with risk of breast cancer in Discovery cohort by multivariate logistic regression model

Genotype	rs11245007 (C/T)			
	CC	CT	TT	CT + TT
All subjects				
Cases (n = 1532)	432 (28.20)	738 (48.17)	362 (23.63)	1100 (71.80)
Controls (n = 1573)	443 (28.16)	768 (48.82)	362 (23.01)	1130 (71.84)
Crude OR (95 % CI)	1.00	0.985 (0.834,1.164)	1.025 (0.842, 1.249)	0.998 (0.854, 1.167)
*P*		0.863	0.802	0.982
Adjusted OR (95 % CI)^a^	1.00	0.796 (0.638,0.992)	0.952 (0.735,1.235)	0.844 (0.687,1.037)
*P*		**0.0420**	0.7124	0.1069
Parity <3^b^				
Cases (n = 1293)	347 (26.84)	636 (49.19)	310 (23.98)	946 (73.16)
Controls (n = 1122)	314 (27.99)	564 (50.27)	244 (21.75)	808 (72.01)
Adjusted OR (95 % CI)^a^	1.00	0.962 (0.751,1.232)	1.324 (0.987,1775)	1.066 (0.846,1.345)
*P*		0.7605	0.0607	0.5865
Parity ≥3^b^				
Cases (n = 188)	67(35.64)	81 (43.09)	40 (21.28)	121 (64.36)
Controls (n = 426)	119 (27.93)	194 (45.54)	113 (26.53)	307 (72.07)
Adjusted OR (95 % CI)^a^	1.00	0.517 (0.304,0.879)	0.469 (0.251,0.876)	0.499 (0.307,0.813)
*P*		**0.0148**	**0.0175**	**0.0053**
Family history of cancer^c^				
Cases (n = 474)	126 (26.58)	238 (50.21)	110 (23.21)	348 (73.42)
Controls (n = 169)	31 (18.34)	95 (56.21)	43 (25.44)	138 (81.66)
Adjusted OR (95 % CI)^a^	1.00	0.530 (0.299,0.937)	0.592 (0.304,1.151)	0.548 (0.317,0.946)
*P*		**0.0289**	0.1223	**0.0308**
No family history of cancer^c^				
Cases (n = 1057)	306 (28.95)	500 (47.30)	251 (23.75)	751 (71.05)
Controls (n = 1401)	411 (29.34)	672 (47.97)	318 (22.70)	990 (70.66)
Adjusted OR (95 % CI)^a^	1.00	0.919 (0.723,1.166)	1.130 (0.854,1.494)	0.985 (0.788,1.230)
*P*		0.4860	0.3933	0.8908

Abbreviations: *OR* Odds ratios; *CI* confidence interval

^a^OR is adjusted for age, BMI, menarche age, parity, time of breast feeding, menopause, oral contraception, smoking status (ever/never), exercise, benign breast disease, and family history of cancer (first- and second-degree relatives)

^b^due to missing values, n (total cases with parity data) < 1 532, n (total controls with parity data) < 1 573

^c^due to missing values, n (total cases with family history of cancer data) < 1 532, n (total controls with family history of cancer data) < 1 573

However, when the results were further stratified by parity (birth number), we found that both the rs11245007 CT and TT genotypes were associated with a decreased risk of breast cancer in subjects who had given birth more than three times, compared with the CC genotype. When combined together, the protective effect of the T allele (CT + TT genotype) was more pronounced in subjects with parity > =3 (OR, 0.499; 95 % CI, 0.307-0.813; *P* = 0.0053) (Table 1). For those with the family history of cancer (positive family history of cancer in first- and second-degree relatives), the CT genotype was also associated with a decreased breast cancer risk (OR, 0.530; 95 % CI, 0.299-0.937; *P* = 0.0289), compared with the CC genotype. Paradoxically, the TT genotype was not significantly associated with a decreased breast cancer risk in women with family history (OR, 0.592; 95 % CI, 0.304-1.151; *P* = 0.1223) (Table 1).

To verify our findings that rs11245007 modulated breast cancer risk in women with parity ≥3 and clarify the effect of rs11245007 on breast cancer risk in women with family history, we further perform an independent case–control analysis (Replication cohort). The subjects in this cohort were recruited from Guangzhou, a major city in South China where is 1 493 miles away from Tianjin, excluding the probability of repetitive recruitment (Additional file 4). Some differences from the discovery set in the distributions of the aforementioned baseline variables between cases and controls were observed in the validation set, even that the cases had a higher percentage of known breast cancer factors, such as family history of cancer and menstruation. These possibly resulted from the relatively smaller sample number of this cohort (just over half of the discovery cohort). The controls in this cohort showed a higher proportion of early menarche (10.8 vs. 4.21 %) and infertility (6.70 % vs. 1.34 %) than those in the discovery cohort. indicating some selection bias in controls of the replication set as well as the geographic variance in economy and culture. Consistently, there were no significant associations between the rs11245007 genotypes and patients clinical features including lymph node metastasis and expression status of ER or PR in Replication cohort (Additional file 5).

In concert with results of the case–control study in Discovery cohort (Table 1), the rs11245007 genotypes were not significantly associated with an overall protective effect on breast cancer risk in Replication cohort. Remarkably, the protective effect of the 905 T allele (CT + TT) on breast cancer risk was still statistically significant in subjects with parity ≥3 (adjusted OR = 0.558; 95 % CI: 0.363– 0.857; *P* = 0.0077), and such protective effect was significant for CT (adjusted OR = 0.547, 95 % CI: 0.343– 0.872; *P* = 0.0113) and TT (adjusted OR = 0.579, 95 % CI: 0.336– 0.997; *P* = 0.0492) genotypes in triparous women (Table 2). The association between rs11245007 genotypes and family history of cancer in Replication cohort was not statistically significant (Table 2).

**Table 2:** Overall and stratified analyses on the association of rs11245007 genotypes with risk of breast cancer in Replication cohort by multivariate logistic regression model

Genotype	rs11245007 (C/T)			
	CC	CT	TT	CT + TT
All subjects				
Cases (n = 804)	243 (30.22)	364 (45.27)	197 (24.50)	561 (69.78)
Controls (n = 835)	223 (26.71)	392 (46.95)	220 (26.35)	612 (73.29)
Crude OR (95 % CI)	1.00	0.852 (0.676,1.074)	0.822 (0.631,1.071)	0.841 (0.679,1.043)
*P*		0.1748	0.1460	0.1148
Adjusted OR (95 % CI)^a^	1.00	0.863 (0.668,1.115)	0.789 (0.587,1.060)	0.836 (0.659,1.061)
*P*		0.2601	0.1158	0.1405
Parity <3^b^				
Cases (n = 468)	129 (27.56)	218 (46.58)	121 (25.85)	339 (72.44)
Controls (n = 505)	137 (27.13)	230 (45.54)	138 (27.33)	368 (72.87)
Adjusted OR (95 % CI)^a^	1.00	1.059 (0.774,1.449)	0.922 (0.646,1.318)	1.008 (0.753,1.349)
*P*		0.7209	0.6571	0.9590
Parity ≥3^b^				
Cases (n = 234)	92 (39.32)	93 (39.74)	49 (20.94)	142 (60.68)
Controls (n = 224)	62 (27.68)	107 (47.77)	55 (24.55)	162 (72.32)
Adjusted OR (95 % CI)^a^	1.00	0.547 (0.343,0.872)	0.579 (0.336,0.997)	0.558 (0.363,0.857)
*P*		**0.0113**	**0.0492**	**0.0077**
Family history of cancer				
Cases (n = 81)	23 (28.40)	33 (40.74)	25 (30.86)	58 (71.60)
Controls (n = 56)	11 (19.64)	27 (48.21)	18 (32.14)	45 (80.36)
Adjusted OR (95 % CI)^a^	1.00	0.477 (0.179,1.270)	0.390 (0.134,1.135)	0.440 (0.178,1.089)
*P*		0.1384	0.0839	0.0759
No family history of cancer				
Cases (n = 723)	220 (30.43)	331 (45.78)	172 (23.79)	503 (69.57)
Controls (n = 779)	212 (27.21)	365 (46.85)	202 (25.93)	567 (72.79)
Adjusted OR (95 % CI)^a^	1.00	0.903 (0.691,1.181)	0.836 (0.612,1.141)	0.879 (0.685,1.128)
*P*		0.4569	0.2585	0.3115

Abbreviations: *OR* Odds ratios, *CI* confidence interval

^a^OR is adjusted for age, BMI, menarche age, parity, menopause, smoking status (ever/never), and family history of cancer (first- and second-degree relatives)

^b^due to missing values, n (total cases with parity data) < 804, n (total controls with parity data) < 835

Considering the sampling error and random error due to geographic difference between these two cohorts, meta-analyses were used to evaluate the potential heterogeneity between these two cohorts and to calculate the pooled association between SNP and breast cancer risk with ORs from these two cohorts (shown in Additional file 6). As expected, there were no heterogeneities observed in the two cohorts, for both the overall and stratified analysis on the association between *FATS* SNP genotypes and breast cancer risk (all *P* > 0.10). Genotypes with T-allele (CT + TT genotype) was associated with a decreased overall risk of breast cancer compared with the CC genotype (OR = 0.84; 95 % CI: 0.71– 0.97). Further, this protective effect of the T allele was more pronounced in subjects with parity > =3 (OR = 0.53; 95 % CI: 0.35– 0.71) and with family history of cancer (OR = 0.51; 95 % CI: 0.25– 0.77).

## Discussion

In this study, we first revealed that a SNP rs11245007, which is located in the largest exon of FATS, is functional in facilitating p53 activation. Two independent case–control studies validated that rs11245007 is an important genetic variant affecting the susceptibility to parity-dependent breast cancer.

Breast tissues undergo extensive physiologic changes during full-term pregnancy, which may vulnerable to breast carcinogenesis. Two of the earliest known and most reproducible features of breast cancer epidemiology are the decreased risk associated with parity and early age at first full-term pregnancy. The long-term protective effect of a term birth on breast cancer risk is preceded by a short-term adverse effect, possibly reflecting a promoting effect of pregnancy hormones. The short-term adverse effect of parity on breast cancer risk is much more evident in women with parity ≥3 and without family history of cancer [7]. Although differentiation of breast cells after the first full-term birth makes them less susceptible to hormonal stimuli, it may not prevent a promoting/progressive effect of pregnancy hormones on breast cells that may be in the very early stages of a carcinogenic process [20]. The high risk after higher order births may be result from a “carry-over” effect of a previous birth. Despite the extensive and productive research on genetic variants and association with overall breast cancer risk, the regulatory pathway underlying parity-associated breast carcinogenesis and the genetic variants conferring susceptibility to parity-dependent risk of breast cancer remain poorly understood.

Recent data support the idea that the crosstalk between FATS and p53 may be of physiological importance for reproduction during evolution. The expression of FATS mRNA and protein is highest in testis and its protein level is second highest in ovary of mouse [10, 11]. Interestingly, our previous data also found the relevance of deficient FATS expression to the onset of breast cancer [14]. Likewise, p53 is a guardian of maternal reproduction. The sufficient expression of p53 is important not only for the implantation of fertilized eggs and prevention of preterm birth in mice, but also for the resistance to pregnancy-related mammary carcinogensis in mice [3, 21]. These facts suggest that the positive feedback loop between FATS and p53 may be indispensable for tighter surveillance of genomic stability during reproduction-related physiological processes. The relevance of deficient FATS expression to the onset of breast cancer and the role of p53 as a guardian of maternal reproduction support our findings that the promotion of p53 activation by *FATS* is important to inhibit pregnancy-related mammary carcinogenesis. Thus, genetic polymorphisms, especially SNPs in *FATS*, those potentially functional to the activation of p53 by FATS may affect the susceptibility to breast cancer.

Interestingly, *FATS* rs11245007 (262D/N) variant, regulating p53 function, did not affect the overall risk of breast cancer in our study population. Similarly, the genetic variants in *Tp53* do not show effect on the risk of breast cancer [22], suggesting that any effect of genetic variants in *Tp53* or *FATS* on breast cancer would be very small or possibly confined to subgroups. Indeed, although *BRCA1*, *BRCA2* and *TP53* mutations confer susceptibility to breast cancer, common variants in these genes have not been shown to confer measurably increased risks of breast cancer [23]. Unexpectedly, we found that the effect of *FATS* rs11245007 variant on breast cancer risk was confined to women with parity ≥3. Both the rs11245007 CT and TT genotypes were associated with a decreased risk of breast cancer in subjects who had given birth more than three times as compared with the CC genotype, in both the Discovery and Replication cohorts. The stronger activation of p53 mediated by FATS 262 N would be physiologically important to suppress carcinogenesis of breast tissues undergoing repetitive and extensive changes during pregnancy for triparous women, and even a small protective effect of FATS-p53 signaling cascade may contribute significantly to decrease the risk of breast cancer as parity increases. The protective effect of FATS-p53 signaling cascade on breast cancer risk may be confined to the subgroup of triparous women. Although rs11245007 T allele (CT + TT) in *FATS* conferred a reduced risk of breast cancer in individuals with a family history of cancer in Tianjin Discovery cohort, such effect was not replicated in the Replication set of Guangzhou cohort, possibly due to the relatively smaller number of the Replication cohort, particularly for the stratification analysis.

## Conclusions

In summary, we have identified for the first time a genetic variant in the *FATS* gene (905C > T, 262D/N) that is associated with susceptibility to breast cancer in a parity-dependent manner. Functional analysis demonstrated that FATS-262 N significantly increased the p53 activity in breast cells, resulting from more pronounced polyubiquitination of p53 by FATS-262 N. These findings provide the emerging physiologic evidence in support of the role of FATS as an E2-independent E3 toward p53, in addition to pinpointing a genetic marker with potential value in predicting breast cancer risk in women with parity ≥3. Once this genetic variant is validated by larger studies in different ethnicities, *FATS* genotyping for 905C > T may have application in breast cancer prevention.

## Additional files

Additional file 1: **Selection for candidate FATS SNPs with the minor allele frequency (MAF) > 0.01 reported within the 1.0 kb promoter region, 5’-UTR, coding region, and 3’-UTR of FATS gene.** (DOCX 17 kb)
Additional file 2: **Baseline characteristics of breast cancer cases and cancer-free controls in Discovery cohort.** (DOCX 18 kb)
Additional file 3: **Frequency distributions of FATS rs11245007 genotypes according to clinical characteristics of cases in Discovery cohort.** (DOCX 18 kb)
Additional file 4: **Baseline characteristics of breast cancer cases and cancer-free controls in Replication cohort.** (DOCX 20 kb)
Additional file 5: **Frequency distributions of** ***FATS*** **rs11245007 genotypes according to clinical characteristics of cases in Replication cohort.** (DOCX 18 kb)
Additional file 6: **Forest plots describing the association between the** ***FATS*** **SNP (A: Genotype CT vs. CC; B: Genotype TT vs. CC; C: Genotype CT + TT vs. CC) and risk of breast cancer from the Discovery and Replication cohorts.** Heterogeneity from the two cohorts was estimated by the *I*^2^, and a fixed-effects model (Mantel-Hansel method) was used to calculate the pooled OR in the absence (all *P* > 0.10) of heterogeneity. (DOCX 229 kb)

## Abbreviations

FATS: Fragile-site associated tumor suppressor
CFS: Common fragile site
SNP: Single nucleotide polymorphism
MAF: Minor allele frequency
STR: Short tandem repeat
ORs: Odds ratios
Cis: Confidence intervals
ER: Estrogen receptor
PR: Progesterone receptor
