# Supplementary material for: A functional genetic variant in fragile-site gene FATS modulates the risk of breast cancer in triparous women
Source: BMC Cancer. 2015 Jul 30;15:559. doi: 10.1186/s12885-015-1570-9 (PMC4520099; doi:10.1186/s12885-015-1570-9)
Supplement: Additional file 1: — Selection for candidate FATS SNPs with the minor allele frequency (MAF) > 0.01 reported within the 1.0 kb promoter region, 5’-UTR, coding region, and 3’-UTR of FATS gene. (DOCX 17 kb) [file 12885_2015_1570_MOESM1_ESM.docx]

| Location | SNP | Alleles | Amino acid position | MAF in database | Function |
| --- | --- | --- | --- | --- | --- |
| 5’-UTR | rs4962342 | G/T | / | 0.109 | 5’-UTR |
| Exon 2 | rs1436803 | A/G | 32 | 0.124 | synonymous |
| Exon 3 | rs11245008 | C/T | 134 | 0.215 | missense |
| Exon 3 | rs78112769 | C/T | 153 | 0.017 | missense |
| Exon 3 | rs11245007 | C/T | 262 | 0.499 | missense |
| Exon 3 | rs78267894 | C/G/T | 272 | 0.016 | synonymous |
| Exon 3 | rs114071782 | A/T | 392 | 0.013 | synonymous |
| Exon 3 | rs73373045 | A/G | 395 | 0.015 | synonymous |
| Exon 4  Exon 4 | rs74814928  rs114882604 | A/G  A/G | 457  472 | 0.016  0.012 | synonymous  nonsense |
| Exon 5 | rs12412320 | G/T | 531 | 0.122 | nonsense |
| 3’-UTR | rs3812676 | C/T | / | 0.238 | 3’-UTR |

**Additional file 1. Selection for candidate *FATS* SNPs with the minor allele frequency (MAF) > 0.01 reported within the 1.0 kb promoter region, 5’-UTR, coding region, and 3’-UTR of *FATS* gene**
