# Supplementary material for: A functional genetic variant in fragile-site gene FATS modulates the risk of breast cancer in triparous women
Source: BMC Cancer. 2015 Jul 30;15:559. doi: 10.1186/s12885-015-1570-9 (PMC4520099; doi:10.1186/s12885-015-1570-9)
Supplement: Additional file 2: — Baseline characteristics of breast cancer cases and cancer-free controls in Discovery cohort. (DOCX 18 kb) [file 12885_2015_1570_MOESM2_ESM.docx]

**Additional file 2. Baseline characteristics of breast cancer cases and cancer-free controls in Discovery cohort**

| **Variables** | **n (%)** | | ***P* ^a^** |
| --- | --- | --- | --- |
|  | **Cases (n = 1532)** | **Controls (n = 1573)** |  |
| Age (years) |  |  |  |
| ≤50 | 737 (48.11) | 726 (46.15) | 0.252 |
| >50 | 795 (51.89) | 847 (53.85) |  |
| Menarche age (years) ^c^ |  |  |  |
| ≤ 12 | 149 (9.74) | 66 (4.21) | <0.0001 |
| > 12 | 1380 (90.26) | 1503 (95.79) |  |
| Pregnancy ^c^ |  |  |  |
| Never | 37 (2.44) | 21 (1.34) | 0.00244 |
| Ever | 1481 (97.56) | 1549 (98.66) |  |
| Parity ^c^ |  |  |  |
| <3 | 1293 (87.31) | 1122 (72.48) | <0.0001 |
| ≥3 | 188 (12.69) | 426 (27.52) |  |
| Breast-feeding time (months) |  |  |  |
| ≤12 | 727 (47.45) | 583 (37.06) | <0.0001 |
| >12 | 805 (52.55) | 990 (62.94) |  |
| Menopause ^c^ |  |  |  |
| No | 727 (47.67) | 713 (45.62) | 0.2525 |
| Yes | 798 (52.33) | 850 (54.38) |  |
| Oral contraception ^c^ |  |  |  |
| Never | 1185 (81.50) | 1294 (83.97) | 0.0734 |
| Ever | 269 (18.50) | 247 (16.03) |  |
| Exercise (times/week) ^c^ |  |  |  |
| ≤1 | 1403 (95.31) | 1448 (92.76) | <0.0031 |
| >1 | 69 (4.69) | 113 (7.24) |  |
| Benign breast disease ^c^ |  |  |  |
| Never | 1120 (73.64) | 1450 (93.19) | <0.0001 |
| Ever | 401 (26.36) | 106 (6.81) |  |
| Family history of cancer ^b, c^ |  |  |  |
| No | 1057 (69.04) | 1401 (89.24) | <0.0001 |
| Yes | 474 (30.96) | 169 (10.76) |  |

**^a^** Two-sided χ^2^ test.

**^b^** First- and second-degree of relatives.

**^c^** due to missing values, n(case) < 1 532, n(control) < 1 573.
