# Supplementary material for: A functional genetic variant in fragile-site gene FATS modulates the risk of breast cancer in triparous women
Source: BMC Cancer. 2015 Jul 30;15:559. doi: 10.1186/s12885-015-1570-9 (PMC4520099; doi:10.1186/s12885-015-1570-9)
Supplement: Additional file 3: — Frequency distributions of FATS rs11245007 genotypes according to clinical characteristics of cases in Discovery cohort. (DOCX 18 kb) [file 12885_2015_1570_MOESM3_ESM.docx]

**Additional file 3. Frequency distributions of *FATS* rs11245007 genotypes according to clinical characteristics of cases in Discovery cohort**

| **Variables** | **n** | **rs11245007 genotype** | | | |
| --- | --- | --- | --- | --- | --- |
|  |  | **CC** | **CT** | **TT** | **CT+TT** |
| Age of diagnosis (mean, S.D.) | 1532 | 51.98 (11.08) | 51.98 (10.91) | 50.87 (10.31) | 51.62 (10.72) |
| *P* **^a^** |  |  | 0.234 |  | 0.551 |
| Lympho nodes metastasis (n, %) | 1513 |  |  |  |  |
| NO |  | 255 (59.72) | 456(62.72) | 218 (60.72) | 674 (62.06) |
| YES |  | 172 (40.28) | 271 (37.28) | 141 (39.28) | 412 (37.94) |
| OR (95% CI) |  | 1.00 | 0.88 (0.69, 1.13) | 0.96 (0.72,1.28) | 0.91 (0.72,1.14) |
| *P* **^b^** |  |  | 0.311 | 0.774 | 0.399 |
| ER (n, %) | 1501 |  |  |  |  |
| - |  | 174 (41.23) | 315 (43.57) | 166 (46.63) | 481 (44.58) |
| + |  | 248 (58.77) | 408 (56.43) | 190 (53.37) | 598 (55.42) |
| OR (95% CI) |  | 1.00 | 0.91 (0.71, 1.16) | 0.80 (0.60,1.07) | 0.87 (0.69,1.10) |
| *P* ^b^ |  |  | 0.441 | 0.131 | 0.240 |
| PR (n, %) | 1501 |  |  |  |  |
| - |  | 189 (44.89) | 302 (41.77) | 167 (46.78) | 469 (43.43) |
| + |  | 232 (55.11) | 421 (58.23) | 190 (53.22) | 611 (56.57) |
| OR (95% CI) |  | 1.00 | 1.14 0.89, 1.45) | 0.93 (0.70,1.23) | 1.06 (0.85,1.33) |
| *P* **^b^** |  |  | 0.304 | 0.599 | 0.606 |

Abbreviations: OR, Odds ratios; CI, confidence interval; ER, estrogen receptor; PR, progesterone receptor.

^a^ Two-sided T test.

^b^ unconditional univariate logistic regression analysis.
