# Supplementary material for: A functional genetic variant in fragile-site gene FATS modulates the risk of breast cancer in triparous women
Source: BMC Cancer. 2015 Jul 30;15:559. doi: 10.1186/s12885-015-1570-9 (PMC4520099; doi:10.1186/s12885-015-1570-9)
Supplement: Additional file 4: — Baseline characteristics of breast cancer cases and cancer-free controls in Replication cohort. (DOCX 20 kb) [file 12885_2015_1570_MOESM4_ESM.docx]

**Additional file 4．Baseline characteristics of breast cancer cases and cancer-free controls in** Replication **cohort**

| **Variables** | **n (%)** | | ***P* ^a^** |
| --- | --- | --- | --- |
|  | **Cases (n = 804)** | **Controls (n = 835)** |  |
| Age (years) |  |  |  |
| ≤50 | 491 (61.07) | 481 (57.60) | 0.154 |
| >50 | 313 (38.93) | 354 (42.40) |  |
| Menarche age (years) |  |  |  |
| ≤ 12 | 80 (9.95) | 90 (10.8) | 0.583 |
| > 12 | 724 (90.05) | 745 (89.22) |  |
| Pregnancy ^c^ |  |  |  |
| Never | 30 (4.11) | 49 (6.70) | 0.0284 |
| Ever | 700 (95.89) | 682 (93.30) |  |
| Parity ^c^ |  |  |  |
| <3 | 468 (66.67) | 505 (69.27) | 0.291 |
| ≥3 | 234 (33.33) | 224 (30.73) |  |
| Breast-feeding time (months) |  |  |  |
| ≤12 | n/a | n/a |  |
| >12 | n/a | n/a |  |
| Menopause ^c^ |  |  |  |
| No | 406 (53.21) | 393 (47.52) | 0.0234 |
| Yes | 357 (46.79) | 434 (52.48) |  |
| Oral contraception |  |  |  |
| Never | n/a | n/a |  |
| Ever | n/a | n/a |  |
| Exercise (times/week) |  |  |  |
| ≤1 | n/a | n/a |  |
| >1 | n/a | n/a |  |
| Benign breast disease |  |  |  |
| Never | n/a | n/a |  |
| Ever | n/a | n/a |  |
| Family history of cancer ^b^ |  |  |  |
| No | 723 (89.93) | 779 (93.29) | 0.0138 |
| Yes | 81 (10.07) | 56 (6.71) |  |

Abbreviations: n/a, not available.

**^a^** Two-sided χ^2^ test.

**^b^** First- and second-degree of relatives.

**^c^** due to missing values, n(case) < 804, n(control) < 835.
