# Supplementary material for: A functional genetic variant in fragile-site gene FATS modulates the risk of breast cancer in triparous women
Source: BMC Cancer. 2015 Jul 30;15:559. doi: 10.1186/s12885-015-1570-9 (PMC4520099; doi:10.1186/s12885-015-1570-9)
Supplement: Additional file 5: — Frequency distributions of FATS rs11245007 genotypes according to clinical characteristics of cases in Replication cohort. (DOCX 18 kb) [file 12885_2015_1570_MOESM5_ESM.docx]

**Additional file 5. Frequency distributions of *FATS* rs11245007 genotypes according to clinical characteristics of cases in Replication cohort**

| **Variables** | **N** | **rs11245007 genotype** | | | | |
| --- | --- | --- | --- | --- | --- | --- |
|  |  | **CC** | **CT** | **TT** | **CT+TT** |  |
| Age of diagnosis (mean, S.D.) | 804 | 49.02 (10.82) | 48.83 (11.28) | 46.75 (11.02) | 48.10 (11.23) |  |
| *P* **^a^** |  |  | 0.0584 |  | 0.2806 |  |
| Lympho nodes metastasis (n, %) | 721 |  |  |  |  |  |
| NO |  | 112 (30.11) | 160(43.01) | 100 (26.88) | 260 (69.89) |  |
| YES |  | 106 (30.37) | 166 (47.56) | 77 (22.06) | 243 (69.63) |  |
| OR (95% CI) |  | 1.00 | 0.97(0.67, 1.42) | 0.65 (0.42,1.02) | 0.85(0.60,1.21) |  |
| *P* **^b^** |  |  | 0.8928 | 0.0592 | 0.3637 |  |
| ER (n, %) | 771 |  |  |  |  |  |
| - |  | 102 (31.29) | 136 (41.72) | 88 (26.99) | 224 (68.71) |  |
| + |  | 132 (29.66) | 213 (47.87) | 100 (22.47) | 313 (70.34) |  |
| OR (95% CI) |  | 1.00 | 1.21 (0.83, 1.75) | 0.95 (0.61,1.46) | 1.11 (0.79,1.57) |  |
| *P* **^b^** |  |  | 0.3272 | 0.8034 | 0.5578 |  |
| PR (n, %) | 804 |  |  |  |  |  |
| - |  | 95 (28.44) | 156 (46.71) | 83 (24.85) | 239 (71.56) |  |
| + |  | 148(31.49) | 208 (44.26) | 114 (24.26) | 322 (68.51) |  |
| OR (95% CI) |  | 1.00 | 0.80(0.55, 1.15) | 0.92 (0.60,1.43) | 0.84 (0.59,1.18) |  |
| *P* **^b^** |  |  | 0.2309 | 0.7222 | 0.3141 |  |

Abbreviations: OR, Odds ratios; CI, confidence interval; ER, estrogen receptor; PR, progesterone receptor.

**^a^** Two-sided T test.

**^b^** unconditional univariate logistic regression analysis.
