# Supplementary material for: A functional genetic variant in fragile-site gene FATS modulates the risk of breast cancer in triparous women
Source: BMC Cancer. 2015 Jul 30;15:559. doi: 10.1186/s12885-015-1570-9 (PMC4520099; doi:10.1186/s12885-015-1570-9)
Supplement: Additional file 6: — Forest plots describing the association between the FATS SNP (A: Genotype CT vs. CC; B: Genotype TT vs. CC; C: Genotype CT + TT vs. CC) and risk of breast cancer from the Discovery and Replication cohorts. Heterogeneity from the two cohorts was estimated by the I2, and a fixed-effects model (Mantel-Hansel method) was used to calculate the pooled OR in the absence (all P > 0.10) of heterogeneity. (DOCX 229 kb) [file 12885_2015_1570_MOESM6_ESM.docx]

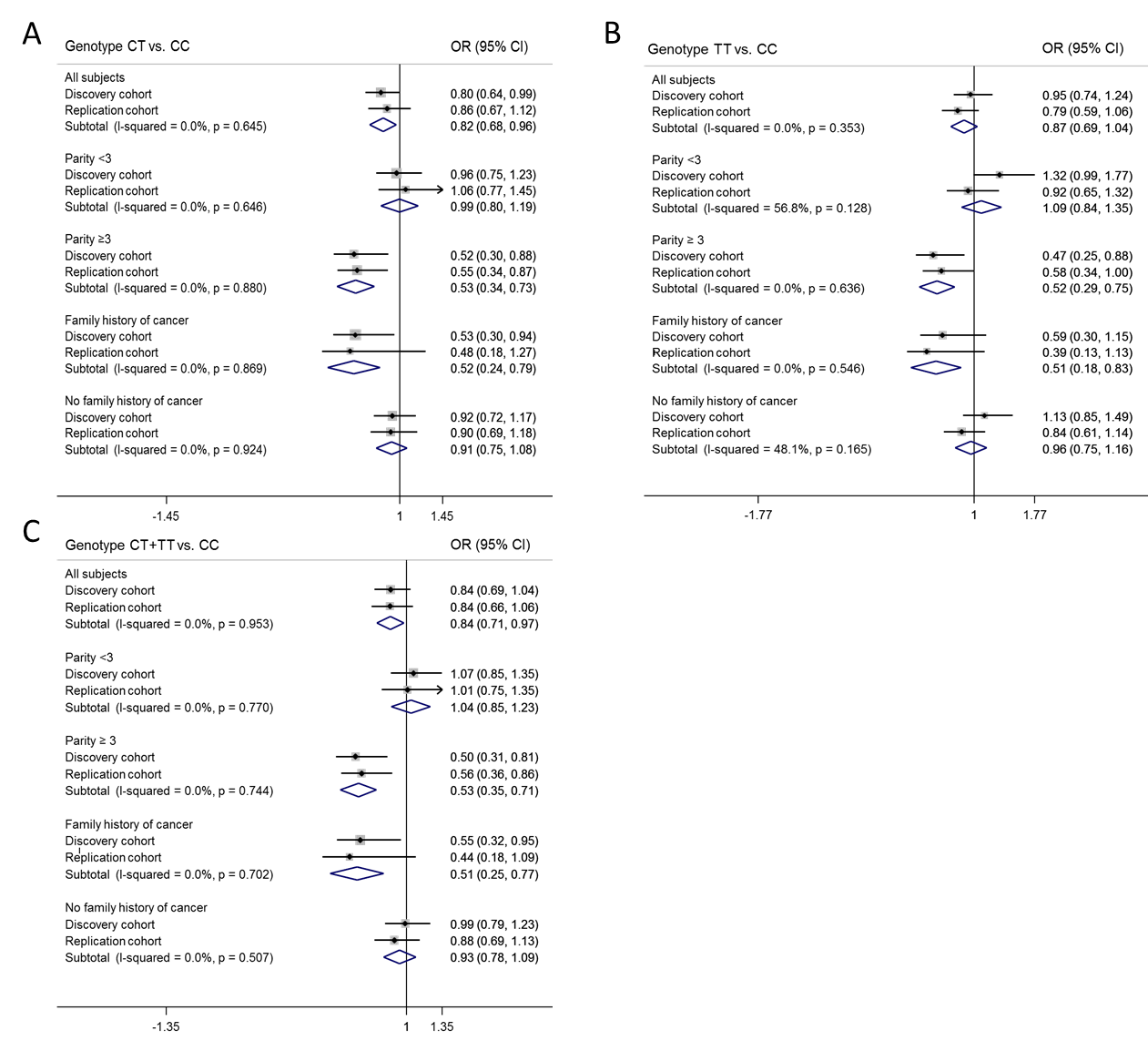


**Additional file 6: Forest plots describing the association between the FATS SNP (A: Genotype CT vs. CC; B: Genotype TT vs. CC; C: Genotype CT+TT vs. CC) and risk of breast cancer from the Discovery and Replication cohorts.** Heterogeneity from the two cohorts was estimated by the *I*^2^, and a fixed-effects model (Mantel-Hansel method) was used to calculate the pooled OR in the absence (all P>0.10) of heterogeneity.
